# Trends in Population-Based Studies of Human Genetics in Infectious Diseases

**DOI:** 10.1371/journal.pone.0025431

**Published:** 2012-02-07

**Authors:** Jessica L. Rowell, Nicole F. Dowling, Wei Yu, Ajay Yesupriya, Lyna Zhang, Marta Gwinn

**Affiliations:** 1 Office of Public Health Genomics, Office of Epidemiology, Surveillance, and Laboratory Services, Centers for Disease Control and Prevention, Atlanta, Georgia, United States of America; 2 National Center for HIV/AIDS, Viral Hepatitis, STD, and TB Prevention, Centers for Disease Control and Prevention, Atlanta, Georgia, United States of America; 3 McKing Consulting Corporation, Atlanta, Georgia, United States of America; Science and Technology Facilities Council, United Kingdom

## Abstract

Pathogen genetics is already a mainstay of public health investigation and control efforts; now advances in technology make it possible to investigate the role of human genetic variation in the epidemiology of infectious diseases. To describe trends in this field, we analyzed articles that were published from 2001 through 2010 and indexed by the HuGE Navigator, a curated online database of PubMed abstracts in human genome epidemiology. We extracted the principal findings from all meta-analyses and genome-wide association studies (GWAS) with an infectious disease-related outcome. Finally, we compared the representation of diseases in HuGE Navigator with their contributions to morbidity worldwide. We identified 3,730 articles on infectious diseases, including 27 meta-analyses and 23 GWAS. The number published each year increased from 148 in 2001 to 543 in 2010 but remained a small fraction (about 7%) of all studies in human genome epidemiology. Most articles were by authors from developed countries, but the percentage by authors from resource-limited countries increased from 9% to 25% during the period studied. The most commonly studied diseases were HIV/AIDS, tuberculosis, hepatitis B infection, hepatitis C infection, sepsis, and malaria. As genomic research methods become more affordable and accessible, population-based research on infectious diseases will be able to examine the role of variation in human as well as pathogen genomes. This approach offers new opportunities for understanding infectious disease susceptibility, severity, treatment, control, and prevention.

## Introduction

Continually evolving human and environmental circumstances—including economic development, increased global travel and commerce, and demographic and behavioral changes—have contributed to the emergence of new infectious diseases and the re-emergence of existing ones [1]. Ever-increasing global connectedness makes control of infectious diseases a global priority. Pathogen genomics has become a leading tool for identifying pathogens, tracking their spread and guiding public health interventions [2], [3].

Rapid advances in molecular technologies and informatics now allow researchers to study human as well as pathogen genetic variation in epidemiologic studies of infectious diseases. During the last decade, population-based research on host genetic factors has extended far beyond the traditional focus of such research on human leukocyte antigens (HLAs) [4]. Although the pace of human gene discovery has been brisk for common chronic diseases and conditions, it has been slower for infectious diseases, which accounted for only 23 of the 978 genome-wide association studies (GWAS) published through 2010. We compiled and analyzed a comprehensive database of published studies in human genome epidemiology (HuGE) of infectious diseases to present a quantitative summary of the field, including its current scope, focus, and trends.

## Methods

To assemble the set of published studies of human genetic associations related to infectious diseases, we used a comprehensive genetic association publications database, the Human Genome Epidemiology (HuGE) Literature Finder (http://www.hugenavigator.net). The HuGE Literature Finder contains articles specifically related to human genome epidemiology, including meta-analyses and systematic reviews, and also allows for filtering of these articles on a number of criteria, including study type (e.g., observational study, meta-analysis), category (e.g., pharmacogenomics, gene-environment interaction), gene, disease, and country of first author. Since 2001, the database has been updated weekly from PubMed (http://www.ncbi.nlm.nih.gov/pubmed) by a combination of automated and human curation procedures [5]. This process has been shown to be highly sensitive (98.5%) and highly specific (97.5%) for retrieval of genetic association articles from PubMed [6].

To identify infectious disease-related articles in the HuGE Literature Finder database, we developed two queries based on medical subject heading (MeSH) terms, which are assigned by PubMed curators (http://www.nlm.nih.gov/mesh). One query used four specific MeSH terms (“bacterial diseases OR mycoses OR virus diseases OR parasitic diseases”) and one used two general MeSH terms (“infectious OR infection”). We compared the performance of the two queries in a subset of articles consisting of those published in 2005 and 2006. Most of the articles identified by the two queries were related to infectious diseases but the results overlapped by only 68%; therefore, we used both queries for our search.

We classified the subset of articles published between 2006 and 2010 (n = 2,456) into five categories based on the relationship between infection and the studied outcome: “infection as primary outcome” (the association of one or more human genetic variants with a specific infectious disease); “infection as a predisposing factor” (genetic susceptibility to a chronic condition given exposure to an infection); “infection as a complication” (genetic susceptibility to infection given a pre-existing chronic condition or predisposing event such as surgery or trauma); “genotype prevalence” (population prevalence of genotypes known to be associated with infectious diseases); and “pharmacogenomics in treatment of infection.”

To check the validity of our outcome classification by category, we selected a random 10% sample of the articles published in 2006–2008 using the RAND() function in Excel version 2007. In addition to the author, two reviewers (M. Gwinn and A. Yesupriya) independently classified the articles in this sample and any disagreements were resolved by discussion among the three reviewers.

We estimated the sensitivity of our combined query for infectious diseases by reviewing the title and abstract of every tenth article excluded by the query for the period from 2006 through 2009. We multiplied by 10 to estimate the numbers of false negative articles (i.e., related to infectious disease but missed by the query) and true negative articles.

All subsequent data analyses were performed with use of SAS version 9. We used a heuristic based on the textbook *Genetic Susceptibility to Infectious Diseases* to classify genes into functional categories (see Table S1) [7]. We used the HuGE Literature Finder filter tool to identify infectious disease-related meta-analyses and extracted the results. We used the National Human Genome Research Institute's Catalog of Published Genome-Wide Association Studies (NHGRI Catalog) to identify all infectious disease-related GWAS published from 2005 through 2010 (including those for diseases with only a suspected infectious origin, such as Kawasaki disease). We extracted GWAS data directly from the NHGRI Catalog, which includes only associations with a reported p level of 1×10^−5^ or lower in the initial GWAS and replication populations, reported either separately or combined [8]. A list of references for all meta-analyses and GWAS are found in the supplementary references.

To assess the alignment of research priorities with public health burden, we examined the correlation of publication frequency (a measure of research output) with disease-specific morbidity for 1) the six most frequently studied infectious diseases; 2) the five most frequently studied health conditions; and 3) the five leading causes of morbidity worldwide. In this analysis, we also included key chronic conditions often associated with one or more of the six most frequently studied infectious diseases: liver cirrhosis, liver cancer (hepatitis B and C infections), and gastric cancer (*H. pylori* infections). The measure of morbidity we used was disability-adjusted life years (DALYs), as calculated for the World Health Organization's *Global burden of disease: 2004 update* [9] (data file available here: http://www.who.int/healthinfo/global_burden_disease/estimates_regional/en/index.html). We chose morbidity instead of mortality because human genetic factors have been studied most frequently in infectious diseases with a chronic course.

## Results

Our queries selected 3,730 articles related to human genetic epidemiology of infectious diseases indexed by the HuGE Navigator from 2001–2010 [10]. The relationship of infection to studied outcomes is summarized in Table 1. Approximately half of the articles focused on infection as the primary outcome; another 20% studied genetic associations with infections as predisposing factors for chronic conditions, such as liver fibrosis or cancer. Approximately 14% of the articles selected by the query were not related to infectious diseases—most often, their abstracts included the keywords “infectious” or “infection” in background sentences describing previous research. Thus, the estimated specificity of our query was 86%.

**Table 1:** Articles related to infectious diseases by studied outcome, HuGE Navigator, 2006–2010.

Article category	n	(%)	Example topics
Infection as primary outcome	1173	(48)	HIV to AIDS progression, malaria susceptibility
Infection as predisposing factor	493	(20)	*H. pylori* and gastric cancer risk, HPV and cervical cancer risk
Pharmacogenomics in treatment of infection	217	(9)	Nevirapine adverse effects in patients with HLA-B*3505allele
Infection as complication	169	(7)	Sepsis after traumatic injury, pneumonia in cystic fibrosis patients
Gene prevalence	64	(3)	Frequency of *CRP* genotypes in ethnic groups in Africa
Not related	340	(14)	multiple sclerosis, chronic fatigue syndrome (where infection not studied)*
Total	2456	(100)	

Classifying a systematic sample of articles according to outcome category, two independent reviewers agreed on 85% (116/137). Through a resolution process, the other 21 articles were finally classified as “Not related [to infectious disease]” (9 articles), “Infection as primary outcome” (6), “Pharmacogenomics in treatment of infection” (2), “Infection as a predisposing factor” (2), or “Infection as a complication” (2).

A systematic 10% sample of articles published in 2006–2009 that were not selected by our query contained 2,722 articles, including 21 articles related to infectious diseases. Thus, we estimated that the query had an overall sensitivity of 89%. The missed (false negative) articles included 10 on periodontitis, 4 on rhinosinusitis, and 2 each on sepsis, leprosy, and tuberculosis. None of these articles included the keywords “infectious” or “infection” in their titles or abstracts. Four missed articles were not indexed with MeSH terms, including one written in a language other than English. The remaining 17 articles were indexed with MeSH terms that referred either to infectious organisms or to clinical conditions such as “sinusitis” that are not cross-referenced with infectious diseases in the MeSH thesaurus.

**Figure 1:**
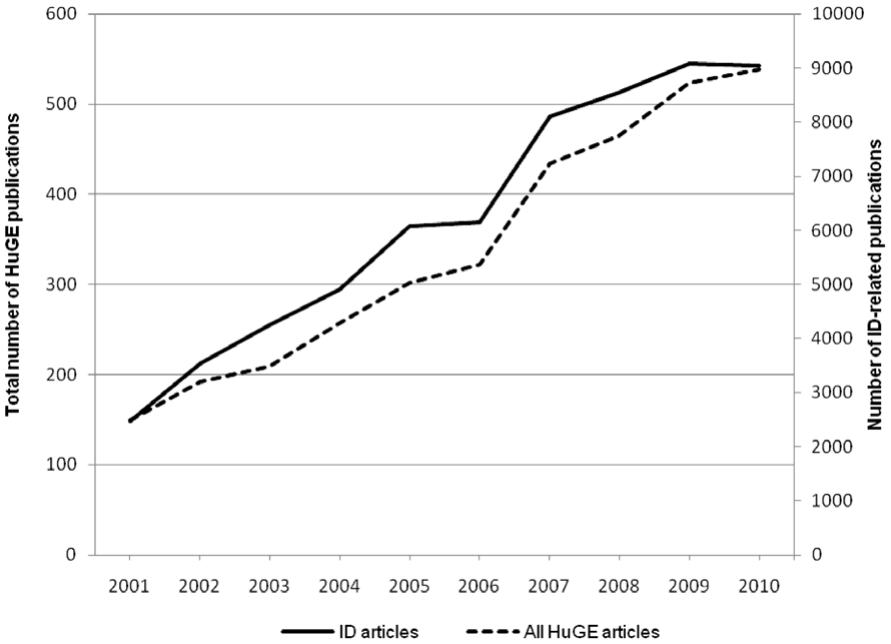
Trend in publication of infectious disease-related articles in human genome epidemiology, 2001–2010.

The annual number of publications related to infectious diseases more than tripled from 148 in 2001 to 543 in 2010; however, the percentage of all articles in the HuGE Literature Finder database that were related to infectious diseases remained nearly constant (range, 5.9–7.3%) (Figure 1). The most commonly studied infectious diseases were human immunodeficiency virus/acquired immune deficiency syndrome (HIV/AIDS, 688 articles), hepatitis C virus (HCV, 410), *Helicobacter pylori* (*H. pylori*) infection (399), tuberculosis (289), hepatitis B virus (HBV, 285), sepsis (254), and malaria (199). Overall, cytokine receptor genes were the most frequently studied category. Among individual genes, *TNF* and *HLA-DRB1* were studied most often, followed by *IL10*, *CCR5*, *IL1B*, and *HLA-B*; each of these genes appeared in more than 200 gene-disease association studies (Tables S2 and S3).

Selected results from the 27 published meta-analyses are summarized in Tables S4, S5, and S6. Six meta-analyses included cohort studies; of these, three were related to HIV/AIDS and were published in 2001 and 2003 (Table S5). Sixteen meta-analyses included only case-control studies (Table S4). Most of these had a combined sample size of more than 2,000 case subjects and more than 2,000 control subjects. The reported odds ratios (ORs) ranged from 1.09 to 2.58 for harmful effects and from 0.90 to 0.12 for protective effects. Of the five meta-analyses related to pharmacogenomics, three included clinical trials (Table S6). Three pharmacogenomics meta-analyses (one on anti-tuberculosis drug-induced hepatotoxicity and two on *H. pylori* eradication) produced statistically significant results, with ORs from 1.73 to 4.28.

**Figure 2:**
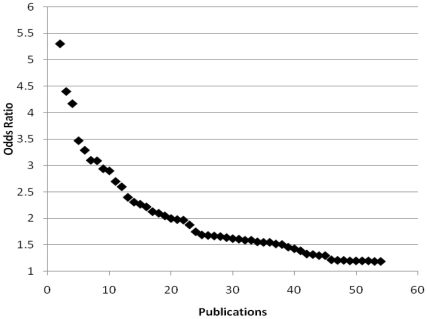
Distribution of effect sizes for infectious disease-related GWAS as reported in the NHGRI Catalog, 2001–2010. One outlying association with an OR of 27.1 (*IL28B* and HCV treatment; Tanaka 2009) is not shown.

Results reported from the 23 infectious disease-related GWAS are summarized in Table S7. Eight of these studies focused on HIV infection or progression to AIDS; four on treatment of HCV infection and viral clearance; one on the role of host genetics in determining susceptibility to atherosclerosis among HIV-infected men on highly active antiretroviral therapy (HAART); and three on chronic diseases with possible infectious agent origins (Kawasaki disease, nasopharyngeal carcinoma, and IgA nephropathy). The other seven GWAS focused on leprosy susceptibility, severe malaria in children, chronic hepatitis B infection, hepatocellular carcinoma (two studies), tuberculosis susceptibility, and meningococcal disease susceptibility. The distribution of effect sizes for significant GWAS results is shown in Figure 2. Approximately one-third of the ORs were between 1.0 and 1.5, one-third were between 1.51 and 2.0, and one-third were greater than 2.0.

**Figure 3:**
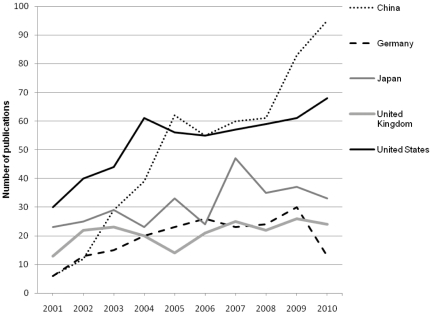
Five countries with the largest numbers of infectious disease-related publications in human genome epidemiology (HuGE), 2001–2010.

The five countries with the most publications from 2001 through 2010 were the United States, China, Japan, United Kingdom, and Germany (Figure 3). Together, they accounted for nearly half of all articles for which the first author's country of residence was known. Five additional countries each produced more than 100 publications during this period: Brazil (158 articles), Italy (157), Spain (140), India (131), and South Korea (103). The most frequently studied diseases varied considerably by country. In China, 145 of 524 (28%) focused on HBV; in the United States, 186 of 576 (33%) focused on HIV/AIDs; and in Japan, 109 of 314 (35%) focused on *H. pylori* infection.

**Figure 4:**
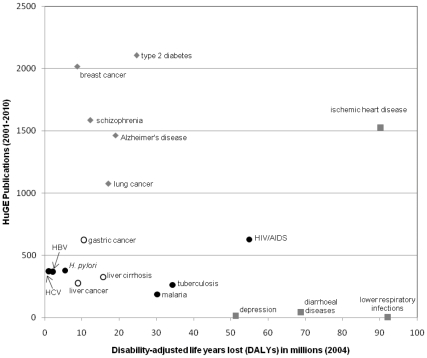
Selected infectious and chronic diseases according to worldwide morbidity (disability-adjusted life-years, DALYs) and publication frequency in human genome epidemiology (HuGE), 2001–2010. Solid black circles represent the six most frequently studied infectious diseases; open black circles represent three chronic diseases that are often part of the natural course of infection with HBV, HCV, or *H. Pylori*. Gray diamonds represent the four most frequently studied diseases overall. Gray squares represent the four diseases with the greatest morbidity worldwide; the fifth is HIV/AIDS, represented with a blue dot. ⊕

Although infectious diseases are leading causes of morbidity worldwide—accounting for 280 million disability-adjusted life years lost (DALYs) annually—they are relatively underrepresented in human genome epidemiologic research (Figure 4). Only HIV/AIDS, which alone accounted for almost 55 million DALYs in 2004, was represented by more than 500 publications during 2001–2010. In contrast, the most frequently studied diseases together accounted for fewer than 82 million disability-adjusted life years (DALYs) lost in 2004.

## Discussion

Since the 19^th^ century, scientists and clinicians have sought explanations for the extensive variation in clinical phenotypes among individuals infected by the same agent. Evidence has been mounting since the 1930s that human genetics may play an important role in this variation [11], [12]. Results from several twin studies support the hypothesis that genetic factors contribute to variations in individual susceptibility to premature death from infectious diseases, as well as to variations in vaccine response [13]. Results of a 2008 study of mortality data for multiple generations of Utah families provided convincing evidence of a heritable predisposition to death from influenza [14].

Results of the Human Genome Project and attendant developments in molecular technology and informatics have enhanced the study of human genetic factors in infectious disease at the population level. The HuGE Navigator database, which has grown rapidly since collection of published studies began in 2001, comprised more than 50,000 articles by the end of 2010. We found that the number of articles related to infectious diseases has increased at roughly the same pace as the total number of articles and still accounts for just 7% of the total.

Research in other fields has laid a strong foundation for exploring the role of human genetics in infectious diseases; in particular, research on immune processes has suggested many candidate genes for further study. We found more than 300 genes whose association with infectious diseases had been studied more than once. The genes most commonly studied were those encoding tumour necrosis factors, cytokine receptors, HLA Class II molecules, and chemokine receptors and their ligands.

Our review of published meta-analyses found many significant genetic associations with infectious diseases; however, statistically significant heterogeneity was found in half the studies that reported testing for it. This heterogeneity could reflect the effects of combining studies that were conducted in populations with different genetic backgrounds (ancestry), or that used different methods for genotyping (selecting and measuring genetic markers) or phenotyping (diagnosing infection and defining clinical outcomes).

The GWAS approach—based on hypothesis-free, systematic genome scanning—has uncovered additional candidate genetic associations with infectious diseases. Some are biologically plausible, such as the association of *IL28B* with spontaneous viral clearance in HCV infection [15]. Others have implicated previously unexplored regions, such as 1p13.3, 9q23, and 8q22.3 in association with AIDS progression [16].

One possible reason for the relative scarcity of infectious disease-related GWAS is the challenge of obtaining large enough study populations with homogeneous phenotypes [17]. For example, the first infectious disease-related GWAS was conducted with 486 HIV-infected patients selected from a potentially eligible group of 30,000 [18]. This study identified two HLA-associated polymorphisms associated with HIV-1 control; however, their replication in an independent cohort did not meet the GWAS Catalog's criterion for statistical significance (p<10^−5^) [8]—probably because the second cohort included only 140 patients. Both associations have been replicated in subsequent GWAS and confirmed by meta-analysis, which increases effective sample size by pooling the results of multiple studies.

Several other approaches have been proposed to discover additional genetic associations relevant to infectious diseases; these include systematic examinations of the entire major histocompatibility (MHC) region and of the set of approximately 1,000 genes involved in innate immunity [19]. Fellay et al. recently suggested an approach for identifying rare variants (not detectable by GWAS) by whole-genome sequencing of a small sample for gene discovery, followed by testing of any associated variants in a larger cohort [20]. Public health surveillance systems offer a potential source of such cohorts [21].

Human genome epidemiologic research on infectious diseases is a global enterprise. We found that the first authors of articles published from 2001–2010 were from 104 countries. Together, the United States, China, Japan, the United Kingdom, and Germany accounted for half of all publications, with China taking the lead after 2007. Our data provide only a minimum estimate of global research output in this field because they are derived from PubMed, which consists mostly of articles written in English.

The most frequently studied infections tended to vary by country, perhaps reflecting these countries' public health priorities. For example, approximately one-third of the articles from the United States, where more than 30,000 new HIV cases have been diagnosed each year since 2005, were related to HIV/AIDS [22]. Nearly one-third of the articles from China, where approximately 8% of people are chronic HBV carriers, focused on HBV infection [23]. Almost half of the articles by Japanese authors focused on health problems related to *H. pylori* infection, which is a significant public health concern in a country where gastric cancer rates are among the highest in the world [24].

HIV/AIDS was the most frequently studied infection and also the largest contributor to global morbidity from infectious diseases (about 55 million DALYs in 2004) [9]. Tuberculosis and malaria each accounted for about 30 million DALYs—nearly twice the number attributed to any of the diseases studied most often for genetic associations (breast cancer, diabetes, Alzheimer's disease, schizophrenia, or lung cancer). In contrast to these adult-onset conditions, infectious diseases affect people of all ages, which accounts in part for their high impact when measured in DALYs.

Although developing countries have the highest rates of morbidity and mortality from infectious diseases, most lack the capacity to conduct human genomics research [25]. In our analysis, Brazil and India ranked among the top 10 countries in numbers of publications; however, 66 of the 104 countries with authors in our database accounted for 10 or fewer articles. Although a few developing countries have built impressive biotechnology infrastructures, most have not, nor have they benefitted from genomic research conducted elsewhere [25]. This lack of participation in genomic research of infectious diseases by countries with high rates of infectious disease indicates a need for a collaborative global effort to support the participation of limited-resource countries in such research. Such collaboration is also important for ethical reasons, so that countries participating in research also share in the benefits [26].

Analysis of pathogen genomics has become a mainstay of public health approaches to surveillance, investigation, and control of infectious diseases. For example, analysis of pathogen restriction-fragment length polymorphism has been used since the 1980s to identify epidemic strains and describe transmission patterns, and genetic changes in influenza viruses are being closely monitored for the emergence of strains with pandemic potential [27]. Researchers are currently investigating the use of additional genomic techniques to improve surveillance of food-borne pathogens [28], [29] and enhance food safety, e.g., by determining safe thresholds of contaminants for vulnerable population sub-groups [29]. Advancements in informatics have led to the development of crucial resources such as continuously updated online databases; one example is the National Center for Biotechnology Information's Entrez Genome database, which contains complete sequence data for more than 1,000 microbes (http://www.ncbi.nlm.nih.gov/sites/entrez).

Studying the role of human genetics in infectious diseases offers new opportunities to understand the etiology and pathology of these diseases by exploring in more depth the determinants of variation in susceptibility, clinical course, and mortality [12]. The path from gene discovery to public health benefit may be more clear-cut for infectious diseases than for many other health conditions [30]; for example, studies of the role of human genetics in infectious diseases have created the new field of vaccinomics, which focuses on predicting vaccine response and avoiding vaccine-related adverse events [31]. Research on both human and pathogen genomes has the potential to identify novel vaccine candidates more quickly than traditional methods of vaccine candidate identification [3]. Better understanding of host-pathogen genome interactions has also encouraged research in innovative therapies to limit and decrease the clinical severity of infections [32].

In our review of human genetic epidemiologic studies since 2001, we found that HIV/AIDS was the most commonly studied infectious disease. The search for human genetic variants that influence HIV infection actually began in the early 1980s, not long after the human immunodeficiency virus was identified. In 2004, Stephen J. O'Brien of the U.S. National Cancer Institute, a pioneer in this research, wrote, “Although AIDS is not generally considered a genetic disease, the considerable heterogeneity in the epidemic is at least partially determined by variants in genes that moderate virus replication and immunity” [33]. CCR5 delta32, discovered in 1996, was only the first of many variants found in epidemiologic cohorts to be associated with HIV infection and AIDS progression. Discovery that an intact CCR5 receptor is an important co-factor in HIV infection has led to targeted drug and vaccine development efforts [33], [34].We found that 7% of articles on genetic associations published from 2001 through 2010 focused on infectious diseases—a disproportionately small fraction, given their public health importance. As genomic research methods become more affordable and accessible, human genome epidemiology will help increase our understanding of people's susceptibility to infectious diseases; the likely severity of these diseases; and how best to prevent, control, and treat them.

## Supporting Information

Table S1 **List of genes by category.** Gene categories based on Kaslow, et al., 2008.
(XLSX)

Table S2 **Gene-disease associations by gene category, 2001–2010.** Gene categories based on Kaslow et al., 2008.
(XLSX)

Table S3 **Number of associations for genes studied at least 50 times, by gene and gene category, 2001–2010.** Gene categories based on Kaslow, et al., 2008.
(XLSX)

Table S4 **Meta-analyses of case-control studies related to infectious diseases, 2001–2010.**
(XLSX)

Table S5 **Meta-analyses of cohort studies related to infectious diseases, 2001–2010.**
(XLSX)

Table S6 **Meta-analyses of pharmacogenomics studies related to infectious diseases, 2001–2010.**
(XLSX)

Table S7 **Genome-wide association studies related to infectious diseases, 2005–2010.**
(XLSX)

Supplementary References S1 **Publications included in Tables S4, S5, S6, S7.**
(DOC)
