# Supplementary material for: Trends in Population-Based Studies of Human Genetics in Infectious Diseases
Source: PLoS One. 2012 Feb 7;7(2):e25431. doi: 10.1371/journal.pone.0025431 (PMC3274513; doi:10.1371/journal.pone.0025431)
Supplement: Supplementary References S1 — Publications included in Tables S4, S5, S6, S7. (DOC) [file pone.0025431.s008.doc]

References for Supplementary Tables S4-S7

1. Alfirevic A, Stalford AC, Vilar FJ, Wilkins EG, Park BK, et al. (2003) Slow acetylator phenotype and genotype in HIV-positive patients with sulphamethoxazole hypersensitivity. Br J Clin Pharmacol 55: 158-165.

2. Auburn S, Diakite M, Fry AE, Ghansah A, Campino S, et al. (2008) Association of the GNAS locus with severe malaria. Hum Genet 124: 499-506.

3. Brouwer MC, de Gans J, Heckenberg SG, Zwinderman AH, van der Poll T, et al. (2009) Host genetic susceptibility to pneumococcal and meningococcal disease: a systematic review and meta-analysis. Lancet Infect Dis 9: 31-44.

4. Brouwer MC, Read RC, van de Beek D (2010) Host genetics and outcome in meningococcal disease: a systematic review and meta-analysis. Lancet Infect Dis 10: 262-274.

5. Burgner D, Davila S, Breunis WB, Ng SB, Li Y, et al. (2009) A genome-wide association study identifies novel and functionally related susceptibility Loci for Kawasaki disease. PLoS Genet 5: e1000319.

6. Chalmers JD, Fleming GB, Hill AT, Kilpatrick DC (2011) Impact of mannose-binding lectin insufficiency on the course of cystic fibrosis: A review and meta-analysis. Glycobiology 21: 271-282.

7. Chauhan M, McGuire W (2008) Interleukin-6 (-174C) polymorphism and the risk of sepsis in very low birth weight infants: meta-analysis. Arch Dis Child Fetal Neonatal Ed 93: F427-429.

8. Clifford RJ, Zhang J, Meerzaman DM, Lyu MS, Hu Y, et al. (2010) Genetic variations at loci involved in the immune response are risk factors for hepatocellular carcinoma. Hepatology 52: 2034-2043.

9. Contopoulos-Ioannidis DG, O'Brien TR, Goedert JJ, Rosenberg PS, Ioannidis JP (2003) Effect of CCR5-delta32 heterozygosity on the risk of perinatal HIV-1 infection: a meta-analysis. J Acquir Immune Defic Syndr 32: 70-76.

10. Davila S, Wright VJ, Khor CC, Sim KS, Binder A, et al. (2010) Genome-wide association study identifies variants in the CFH region associated with host susceptibility to meningococcal disease. Nat Genet 42: 772-776.

11. Feehally J, Farrall M, Boland A, Gale DP, Gut I, et al. (2010) HLA has strongest association with IgA nephropathy in genome-wide analysis. J Am Soc Nephrol 21: 1791-1797.

12. Fellay J, Shianna KV, Ge D, Colombo S, Ledergerber B, et al. (2007) A whole-genome association study of major determinants for host control of HIV-1. Science 317: 944-947.

13. Fellay J, Thompson AJ, Ge D, Gumbs CE, Urban TJ, et al. (2010) ITPA gene variants protect against anaemia in patients treated for chronic hepatitis C. Nature 464: 405-408.

14. Gao L, Tao Y, Zhang L, Jin Q (2010) Vitamin D receptor genetic polymorphisms and tuberculosis: updated systematic review and meta-analysis. Int J Tuberc Lung Dis 14: 15-23.

15. Ge D, Fellay J, Thompson AJ, Simon JS, Shianna KV, et al. (2009) Genetic variation in IL28B predicts hepatitis C treatment-induced viral clearance. Nature 461: 399-401.

16. Hong X, Yu RB, Sun NX, Wang B, Xu YC, et al. (2005) Human leukocyte antigen class II DQB1*0301, DRB1*1101 alleles and spontaneous clearance of hepatitis C virus infection: a meta-analysis. World J Gastroenterol 11: 7302-7307.

17. Ioannidis JP, Contopoulos-Ioannidis DG, Rosenberg PS, Goedert JJ, De Rossi A, et al. (2003) Effects of CCR5-delta32 and CCR2-64I alleles on disease progression of perinatally HIV-1-infected children: an international meta-analysis. AIDS 17: 1631-1638.

18. Ioannidis JP, Rosenberg PS, Goedert JJ, Ashton LJ, Benfield TL, et al. (2001) Effects of CCR5-Delta32, CCR2-64I, and SDF-1 3'A alleles on HIV-1 disease progression: An international meta-analysis of individual-patient data. Ann Intern Med 135: 782-795.

19. Jallow M, Teo YY, Small KS, Rockett KA, Deloukas P, et al. (2009) Genome-wide and fine-resolution association analysis of malaria in West Africa. Nat Genet 41: 657-665.

20. Joubert BR, Lange EM, Franceschini N, Mwapasa V, North KE, et al. (2010) A whole genome association study of mother-to-child transmission of HIV in Malawi. Genome Med 2: 17.

21. Kamatani Y, Wattanapokayakit S, Ochi H, Kawaguchi T, Takahashi A, et al. (2009) A genome-wide association study identifies variants in the HLA-DP locus associated with chronic hepatitis B in Asians. Nat Genet 41: 591-595.

22. Le Clerc S, Limou S, Coulonges C, Carpentier W, Dina C, et al. (2009) Genomewide association study of a rapid progression cohort identifies new susceptibility alleles for AIDS (ANRS Genomewide Association Study 03). J Infect Dis 200: 1194-1201.

23. Lewis SJ, Baker I, Davey Smith G (2005) Meta-analysis of vitamin D receptor polymorphisms and pulmonary tuberculosis risk. Int J Tuberc Lung Dis 9: 1174-1177.

24. Li HT, Zhang TT, Zhou YQ, Huang QH, Huang J (2006) SLC11A1 (formerly NRAMP1) gene polymorphisms and tuberculosis susceptibility: a meta-analysis. Int J Tuberc Lung Dis 10: 3-12.

25. Limou S, Le Clerc S, Coulonges C, Carpentier W, Dina C, et al. (2009) Genomewide association study of an AIDS-nonprogression cohort emphasizes the role played by HLA genes (ANRS Genomewide Association Study 02). J Infect Dis 199: 419-426.

26. Pacheco AG, Cardoso CC, Moraes MO (2008) IFNG +874T/A, IL10 -1082G/A and TNF -308G/A polymorphisms in association with tuberculosis susceptibility: a meta-analysis study. Hum Genet 123: 477-484.

27. Padol S, Yuan Y, Thabane M, Padol IT, Hunt RH (2006) The effect of CYP2C19 polymorphisms on H. pylori eradication rate in dual and triple first-line PPI therapies: a meta-analysis. Am J Gastroenterol 101: 1467-1475.

28. Pelak K, Goldstein DB, Walley NM, Fellay J, Ge D, et al. (2010) Host determinants of HIV-1 control in African Americans. J Infect Dis 201: 1141-1149.

29. Pereira AC, Brito-de-Souza VN, Cardoso CC, Dias-Baptista IM, Parelli FP, et al. (2009) Genetic, epidemiological and biological analysis of interleukin-10 promoter single-nucleotide polymorphisms suggests a definitive role for -819C/T in leprosy susceptibility. Genes Immun 10: 174-180.

30. Pereyra F, Jia X, McLaren PJ, Telenti A, de Bakker PI, et al. (2010) The major genetic determinants of HIV-1 control affect HLA class I peptide presentation. Science 330: 1551-1557.

31. Persson C, Canedo P, Machado JC, El-Omar EM, Forman D (2011) Polymorphisms in inflammatory response genes and their association with gastric cancer: A HuGE systematic review and meta-analyses. Am J Epidemiol 173: 259-270.

32. Petrovski S, Fellay J, Shianna KV, Carpenetti N, Kumwenda J, et al. (2011) Common human genetic variants and HIV-1 susceptibility: a genome-wide survey in a homogeneous African population. AIDS 25: 513-518.

33. Qin H, Liu B, Shi T, Liu Y, Sun Y, et al. (2010) Tumour necrosis factor-alpha polymorphisms and hepatocellular carcinoma: a meta-analysis. J Int Med Res 38: 760-768.

34. Rauch A, Kutalik Z, Descombes P, Cai T, Di Iulio J, et al. (2010) Genetic variation in IL28B is associated with chronic hepatitis C and treatment failure: a genome-wide association study. Gastroenterology 138: 1338-1345, 1345 e1331-1337.

35. Shrestha S, Irvin MR, Taylor KD, Wiener HW, Pajewski NM, et al. (2010) A genome-wide association study of carotid atherosclerosis in HIV-infected men. AIDS 24: 583-592.

36. Sun F, Chen Y, Xiang Y, Zhan S (2008) Drug-metabolising enzyme polymorphisms and predisposition to anti-tuberculosis drug-induced liver injury: a meta-analysis. Int J Tuberc Lung Dis 12: 994-1002.

37. Suppiah V, Moldovan M, Ahlenstiel G, Berg T, Weltman M, et al. (2009) IL28B is associated with response to chronic hepatitis C interferon-alpha and ribavirin therapy. Nat Genet 41: 1100-1104.

38. Tanaka Y, Nishida N, Sugiyama M, Kurosaki M, Matsuura K, et al. (2009) Genome-wide association of IL28B with response to pegylated interferon-alpha and ribavirin therapy for chronic hepatitis C. Nat Genet 41: 1105-1109.

39. Teuffel O, Ethier MC, Beyene J, Sung L (2010) Association between tumor necrosis factor-alpha promoter -308 A/G polymorphism and susceptibility to sepsis and sepsis mortality: a systematic review and meta-analysis. Crit Care Med 38: 276-282.

40. Thye T, Vannberg FO, Wong SH, Owusu-Dabo E, Osei I, et al. (2010) Genome-wide association analyses identifies a susceptibility locus for tuberculosis on chromosome 18q11.2. Nat Genet 42: 739-741.

41. Tse KP, Su WH, Chang KP, Tsang NM, Yu CJ, et al. (2009) Genome-wide association study reveals multiple nasopharyngeal carcinoma-associated loci within the HLA region at chromosome 6p21.3. Am J Hum Genet 85: 194-203.

42. Veloso S, Olona M, Peraire J, Vilades C, Pardo P, et al. (2011) No relationship between TNF-alpha genetic variants and combination antiretroviral therapy-related lipodystrophy syndrome in HIV type 1-infected patients: a case-control study and a meta-analysis. AIDS Res Hum Retroviruses 27: 143-152.

43. Zaffanello M, Tardivo S, Cataldi L, Fanos V, Biban P, et al. (2011) Genetic susceptibility to renal scar formation after urinary tract infection: a systematic review and meta-analysis of candidate gene polymorphisms. Pediatr Nephrol 26: 1017-1029.

44. Zhang FR, Huang W, Chen SM, Sun LD, Liu H, et al. (2009) Genomewide association study of leprosy. N Engl J Med 361: 2609-2618.

45. Zhang H, Zhai Y, Hu Z, Wu C, Qian J, et al. (2010) Genome-wide association study identifies 1p36.22 as a new susceptibility locus for hepatocellular carcinoma in chronic hepatitis B virus carriers. Nat Genet 42: 755-758.

46. Zhang LZ, Zhang TC, Pan FM, Zhang ZH, Li X (2010) Interleukin-10 gene polymorphisms in association with susceptibility to chronic hepatitis C virus infection: a meta-analysis study. Arch Virol 155: 1839-1842.

47. Zhao F, Wang J, Yang Y, Wang X, Shi R, et al. (2008) Effect of CYP2C19 genetic polymorphisms on the efficacy of proton pump inhibitor-based triple therapy for Helicobacter pylori eradication: a meta-analysis. Helicobacter 13: 532-541.

48. Zheng MH, Qiu LX, Xin YN, Pan HF, Shi KQ, et al. (2010) Tumor necrosis factor-alpha-308A allele may have a protective effect for chronic hepatitis B virus infection in Mongoloid populations. Int J Infect Dis 14: e580-585.
